# Bioluminescence Imaging Allows Monitoring Hepatitis C Virus Core Protein Inhibitors in Mice

**DOI:** 10.1371/journal.pone.0014043

**Published:** 2010-11-18

**Authors:** Juan Du, Fang Zhao, Yong Zhou, Hu Yan, Xiang-guo Duan, Sheng-qiang Liang, Ying-li Wang, Qiu-xia Fu, Xiao-hui Wang, Jian-chun Peng, Lin-sheng Zhan

**Affiliations:** 1 Beijing Institute of Transfusion Medicine, Beijing, China; 2 Ningxia Medical University, Yinchuan, China; University Hospital Zurich, Switzerland

## Abstract

**Background:**

The development of small molecule inhibitors of hepatitis C virus (HCV) core protein as antiviral agents has been intensively pursued as a viable strategy to eradicate HCV infection. However, lack of a robust and convenient small animal model has hampered the assessment of in vivo efficacy of any antiviral compound.

**Methodology/Principal Findings:**

The objective of this work was to develop a novel method to screen anti-core protein siRNA in the mouse liver by bioluminescence imaging. The inhibitory effect of two shRNAs targeting the highly conserved core region of the HCV genome, shRNA452 and shRNA523, was examined using this method. In the transient mouse model, the effect of shRNA-523 was detectable at as early as 24 h and became even more pronounced at later time points. The effect of shRNA-452 was not detectable until 48 h post-transduction. In a stable mouse model, shRNA523 reduced luciferase levels by up to 76.4±26.0% and 91.8±8.0% at 6 h and 12 h after injection respectively, and the inhibitory effect persisted for 1 day after a single injection while shRNA-Scramble did not seem to have an effect on the luciferase activity in vivo.

**Conclusions/Significance:**

Thus, we developed a simple and quantitative assay for real-time monitoring of HCV core protein inhibitors in mice.

## Introduction

HCV infection is a major cause of chronic liver diseases, which often progresses to liver cirrhosis and hepatocellular carcinoma (up to 20%) [1]. No vaccine is currently available, and current treatment options involving interferon-α (IFN-α) alone or in combination with ribavirin are ineffective with substantial side effects. Therefore, safer and more efficient therapeutic agents are needed.

HCV is an enveloped RNA virus that belongs to the family Flaviviridae [2].HCV has a single stranded, positive polarity RNA encoding for a polyprotein precursor of about 3000 amino acids, which is further cleaved into 10 mature proteins. The HCV core protein that forms the nucleocapsid is the most conserved protein among the six major HCV genotypes [3], [4]. An immature core protein (p23, residues 1–191) is cleaved by host signal peptide peptidase (SPPase) to generate the mature core protein (p21) within the signal sequence, which is estimated to be between 173 to 181 amino acids in length [5]–[7].The mature core protein plays vital roles in modulating gene transcription, cell proliferation, cell death, oxidative stress, and immunomodulation in host cells [8]–[12]. Small molecule inhibitors of HCV core protein as antiviral agents have been under intensive development as a viable strategy to eradicate HCV infection, yet lack of a robust and convenient small animal model has hindered the assessment of in vivo efficacy of any antiviral compounds.

In the present work, we established a transient mouse model and stable mouse model by hydrodynamics methods to screen of HCV core protein inhibitors. The inhibitory effect of hairpin shRNAs targeting the core region of the HCV genome was monitored in the mouse liver by bioluminescence imaging. Finally, we found that the expression level of core protein could be reflected by the activity of Fluc in the mouse model, and shRNA targeting HCV core protein could effectively downregulate core gene and Fluc gene expression in vivo. These models could be used for screening anti-HCV compounds.

## Materials and Methods

### Mice

C57BL/6 mice (male, 4–6 weeks) were obtained from and fed in National Beijing Center for Drug Safety Evaluation and Research (NBCDSER).This study was approved by the ethics committee of the NBCDSER (Permit No.09-1425).

### Plasmids construction

**Figure 1:**
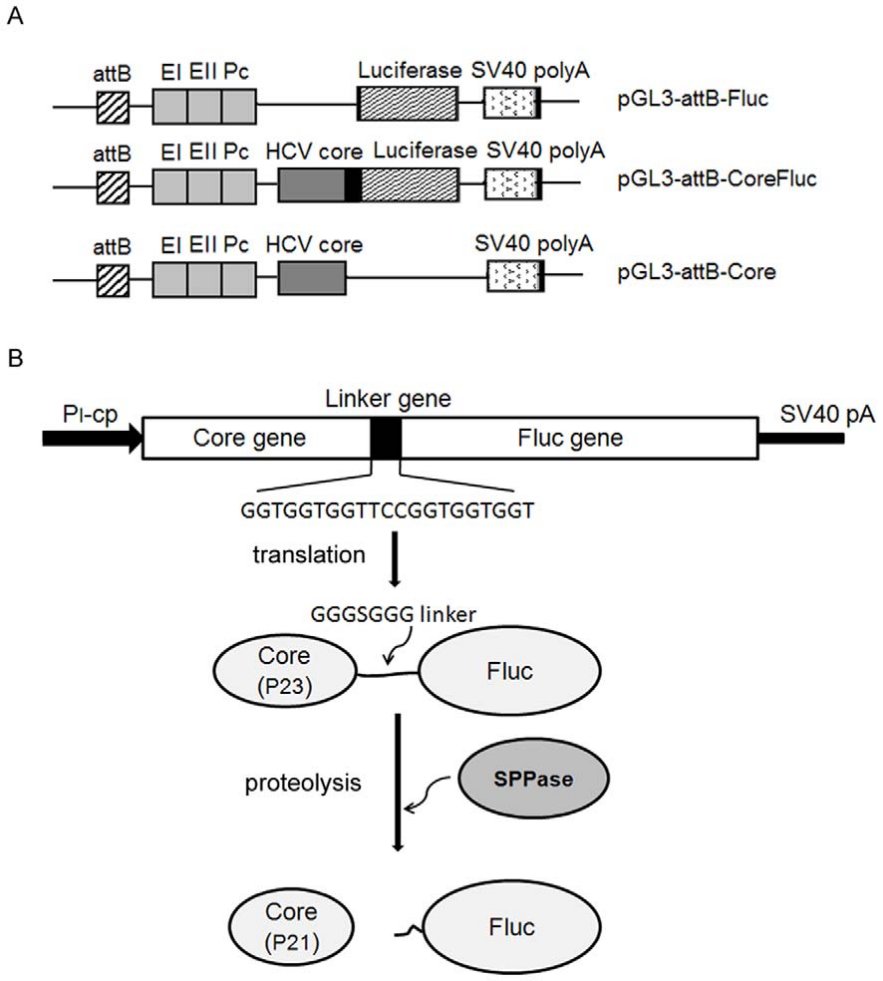
Schematic diagram of plasmid and processing of the Core-Fluc fusion protein. A.Schematic representation of the plasmids used in the present study. B. Schematic diagram of Core-Fluc fusion protein processing. pGL3-*attB*-CoreFluc plasmid encoded a precursor protein which was composed of the entire core protein (aa 1–191) and Fluc protein and was further processed into two proteins (the mature core protein (p21) and Fluc) within the signal sequence by host SPPase [15].Thus the expression level of core protein was reflected by the activity of Fluc which could be detected by the IVIS camera in whole animals.

pCMVInt and pT-*attB*, as were previously described [13], were kindly provided by M. P. Calos, Department of Genetics, Stanford University, USA. Plasmid pGL3-*attB*-Fluc (Fig. 1A) was generated by cloning a 297-bp fragment from pT-*attB* containing the minimal length ФC31 *attB* site and surrounding sequence into the *Kpn* I site of pGL3-EI-EII-Pc [14]. For the generation of pGL3-*attB*-CoreFluc, HCV core sequence was amplified by PCR using clone Con1 as the template and was cloned into the *Nco* I site upstream of the firefly luciferase gene of pGL3-*attB*-Fluc.In pGL3-*attB*-CoreFluc, the firefly luciferase (FLuc) gene was fused in-frame to the downstream of HCV core gene by a short linker gene (GGTGGTGGTTCCGGTGGTGGT). Plasmid pGL3-*attB*-Core was generated by deleting the Fluc gene from the pGL3-*attB*-CoreFluc between NcoI and XbaI sites. The plasmids expressing shRNAs against the following regions of the HCV core-protein sequence: 452–472 nt, 479–499 nt, and 503–523 nt, were also constructed [16]–[18]. Sense and antisense strands of shRNA oligonucleotides were synthesized, annealed and cloned into the BamHI and HindIII sites of pSilencer-2.1-U6 neo plasmid (Ambion, Austin, Texas ). Scrambled shRNA (control shRNA) cloned into the same vector was used as a negative control.

### Cell cultures, DNA transfections, and luciferase assays

Huh7 cells were cultured in DMEM (Gibco, Carlsbad, CA) with 10% FBS (HyClone, South Logan,UT) at 37°C in 5% CO2/air. Transient transfections were performed by using Lipofectamine 2000 (Invitrogen, Carlsbad, CA), starting from approximately 1×10^5^ cells/well in 24-well dishes with 0.6 µg of a plasmid DNA mixture following the manufacturer's instructions. Plasmids were purified with the Qiagen plasmid purification kits (Hilden, Germany). In each case, 1 ng of a plasmid that had the *Renilla* luciferase gene driven by the herpes simplex virus thymidine kinase (HSV-TK) promoter (pRL-TK, Promega) was included in the assay to monitor transfection efficiency. After 48 h, cells were washed with PBS and harvested in 100 µl of Passive Lysis Buffer (PLB; Promega). *Firefly* and *Renilla luciferase* activity was measured in a GloMax™ 96 luminometer from 20 µl of lysate using the Dual-Luciferase Reporter Assay System (Promega).

### In vivo gene delivery and determination of luciferase expression in the mouse liver

For the long-term study, plasmids were purified with the Endotoxin Free Maxi Kit (Qiagen, Hilden, Germany) and administrated to C57BL/6 mice by the hydrodynamics method [19], [20]. Three C57BL/6 mice were used in each group. Ten micrograms of DNA mixture in 1.6 ml saline was intravenously injected in a time range of 5to 8 s. Animals were imaged in the Xenogen IVIS-50 optical imaging system at the indicated time described in the article.

### Isolation of livers and analysis of genomic integration by Nested PCR

Animals were sacrificed after 2weeks (control group) and 3 months (test group).The livers were removed and genomic DNA isolated using the Wizard Genomic DNA Purification Kit (Promega) according to the manufacturer's instructions. To detect site specific integration at mpsL1 (mice pseudo-site from liver ), a nested PCR approach was followed. Mice liver genome DNA was used as template for the first round PCR with primers mspL1rev and *attB*-1. The cycling conditions were 94°C for 30 s, 55°C for 30 s and 72°C for 30 s. The products were used as templates in the second round PCR with primers mspL1rev and *attB*-2 under similar conditions to those for the first round PCR. The second-round PCR products were cloned into pGEM-T (Promega) and sequenced. The primers were showed as follows [21]. mspL1rev: 5′-TGAGGAGGAGCCTTAGCAAC-3′; *attB*-1: 5′-GTAGGTCACGGTCTCGAAGC-3′; *attB*-2: 5′-CGAAGCCGCGGTGCGGGTGCCA- 3′.

### Western blot analysis

Proteins were extracted from liver tissues or transfected cells following the manufacturer's instructions. 10 µg of proteins was loaded on a 10% SDS–polyacrylamide gel in Laemmli sample buffer. Proteins were separated by electrophoresis and transferred onto a Hybond-P membrane (Amersham Pharmacia Biotech, England). The membrane was blocked overnight at 4°C in 5% nonfat milk and incubated with anti-HCV-core or anti-luciferase antibody followed by secondary antibody. Immunoreactive proteins were visualized using SuperSignalWest Pico Chemiluminescent Substrate (Pierce).

### Histology

For routine histological analysis, formalin-fixed paraffin-embedded liver samples were cut into sections 4 µm thick, deparaffinized in xylene, and dehydrated through a series of decreasing concentrations of ethanol. Sections were stained with hematoxylin and eosin.

### Serum Alanine Aminotransferase measurement

Serum alanine aminotransferase was measured in 25 µl samples using a microtiter plate assay (Pointe Scientific, Canton, MI)

### Serum IL-6 and IL-1β measurement

IL-6 and IL-1βwere measured by immunoreactivity in a double-sandwich enzyme-linked immunosorbent assay (ELISA) format using commercially available kits for mouse cytokines (RapidBio Lab, California, USA)according to the manufacturer's recommendations.

### Statistics

All data were reported as mean±SD. For statistical comparisons, parametrical data were compared using the Student's t-test. Differences were considered significant at *P*<0.05.

## Results

### Firefly luciferase activity can be used to reflect HCV core protein expression in vitro and in vivo

**Figure 2:**
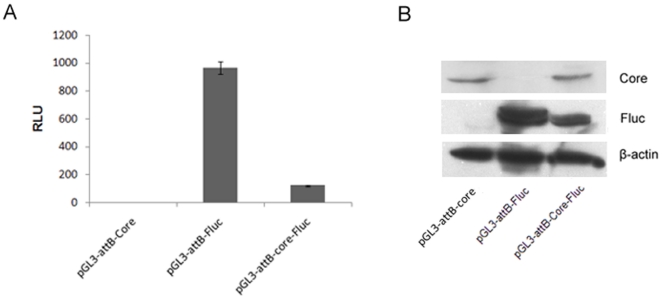
HCV core protein and Fluc expression in Huh 7 cells. A. Fluc expression was detected by Luciferase Assay. The pGL3- *attB*-Core, pGL3-*attB*-Fluc, pGL3-*attB*-Core-Fluc vectors were transfected into Huh-7 cells. After 48 h, the cells were harvested and luciferase activity measured. The transfection efficiency was normalized with *Renilla* luciferase activity and the normalized luciferase activity was plotted. B. Western blot analysis of HCV core protein and Fluc expression. Total cell lysates were prepared from Huh7 cells, and transferred to membranes, followed by incubation with monoclonal antibodies specific for the HCV core (top), Fluc (middle) or β-actin (bottom) that serves as a control for protein loading.

To find out whether these constructs could express core protein and Fluc, pGL3-*attB*-Core, pGL3-*attB*-Fluc and pGL3-*attB*-CoreFluc were transfected into Huh7 cells respectively. Forty-eight hours after transfection, Fluc activities were measured. The Fluc activity was significantly higher in cells transfected with pGL3-*attB*-Fluc than in those transfected with pGL3-*attB*-CoreFluc. No Fluc activity was measured in cells transfected with pGL3-*attB*-Core (Fig. 2A).Total lysates were separated by SDS/polyacrylamide gel electrophoresis and probed for core protein and Fluc as well as β-actin, using specific monoclonal antibodies. As expected, core protein and Fluc were expressed in cells transfected with pGL3-*attB*-CoreFluc, but the expression level of Fluc was lower than that in cells transfected with pGL3-*attB*-Fluc. The expression level of core protein in cells transfected with pGL3-*attB*-CoreFluc was equivalent to that in cells transfected with pGL3-*attB*-Core (Fig. 2B).

**Figure 3:**
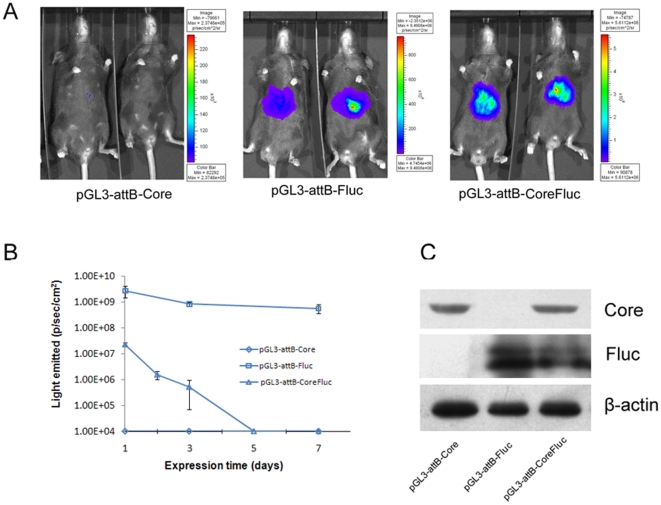
HCV core protein and Fluc expression in the liver of C57BL/6 mice. A. Bioluminescent Imaging analysis of the Fluc activity. C57BL/6 mice were injected with 10 µg pGL3-*attB*-Core, pGL3-*attB*-Fluc, pGL3-*attB*-CoreFluc respectively and monitored by bioluminescent imaging. Fluc activity was measured 24 hours post-injection. B. Evaluation of Fluc activity in vivo over the course of the experiment.Values represent means±S.D. (n = 5). C. Western blot analysis of the HCV core protein and Fluc. Mice were transfected and sacrified at 24 hr. Fluc and core protein detection was performed as described above.

It had been verified that pGL3-*attB*-CoreFluc was successfully expressed in cells. Then we investigated whether these constructs could express core protein and Fluc in the mouse liver. Hydrodynamics-based DNA injection method was employed to deliver genes into C57BL/6 mouse liver. Bioluminescence imaging was performed to examine Fluc activity at different time point after DNA injection. As illustrated in Figure 3A and 3B, the Fluc activity was significantly higher in the livers of mice transfected with pGL3-*attB*-Fluc, approximately two orders of magnitude higher than that in mice transfected with pGL3-*attB*-CoreFluc at 24 h after DNA injection. The transgene expression was transient in pGL3-*attB*-CoreFluc transfected mice, luciferase activity was undetectable beyond day 5. But in pGL3-*attB*-Fluc transfected mice, luciferase activity remained at a high level beyond day7. Western blot analysis was carried out to detect the core protein and Fluc in the mouse liver (Fig. 3C). The expression level of Fluc in mice transfected with pGL3-*attB*-CoreFluc was lower than that in mice transfected with pGL3-*attB*-Fluc. The expression level of core protein in mice transfected with pGL3-*attB*-CoreFluc was equivalent to that in mice transfected with pGL3-*attB*-Core. These results were consistent with the results in cells. All the data suggested that the expression level of core protein determined the Core-Fluc coexpression level in vitro and in vivo.

### Core-shRNA-expression vectors inhibit core protein and Fluc expression in vitro and in vivo

**Figure 4:**
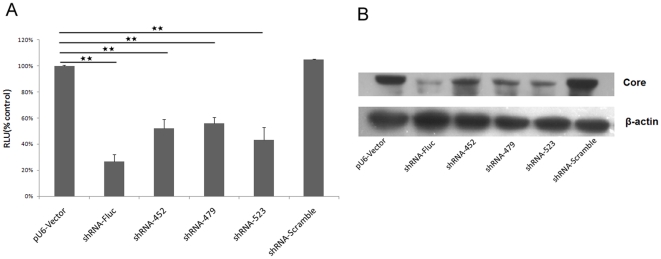
Efficient suppression of the HCV core protein and Fluc expression by pU6- shRNA expression vector in vitro. A.Inhibition of Fluc activity by pU6-mediated shRNA expression vector. shRNA-Scramble vector, shRNA–Fluc vector, shRNA-452 vector, shRNA-479 vector, shRNA-523 vector or pU6-vector (2 µg) were co-transfected with 0.5 µg pGL3-*attB*-CoreFluc vector and pRL-TK into Huh-7 cells. Luciferase reporter assay was performed 48 h post-transfection. The transfection efficiency was normalized with Renilla luciferase activity and the normalized luciferase activity was plotted taking the control (pU6-vector) as 100%. The presence of statistical difference has been indicated as follows: *  =  *p*<0.05; **  =  *p*<0.01. B.Western blot analysis to establish the effects of various shRNA expression vectors on the levels of HCV core protein. Total cell lysates were electrophoresed on 12% SDS polyacrylamide gels, and transferred to membranes, followed by incubation with polyclonal antibodies for the core protein. And β-actin serves as a control for protein loading.

To investigate whether intracellular expression of shRNA inhibited core protein and Fluc expression in cultured cells, three shRNAs(shRNA452, shRNA479, and shRNA523) were designed to target the highly conserved core region of the HCV genome. pGL3-*attB*-CoreFluc and each of the shRNA expression vectors were co-transfected into Huh-7 cells. 48 h after transfection, the cells were lysed and Fluc activities were measured. The results revealed that the luciferase activities were reduced by about 40–55% in the cells co-transfected with shRNA452, shRNA479, and shRNA523 (Fig. 4A). The inhibitory effects of these three shRNAs had no statistical difference. The control-shRNA (scramble-shRNA) had no inhibitory effect. The inhibitory effect of shRNAs was confirmed by western blotting to detect HCV core protein (Fig. 4B). The result showed that core protein expression was reduced in cells co-transfected with Fluc-shRNA, shRNA-452, shRNA-479, or shRNA-523, but not in cells co-transfected with scramble-shRNA. The degradation of HCV core protein was consistent with the reduction of Fluc activity.

**Figure 5:**
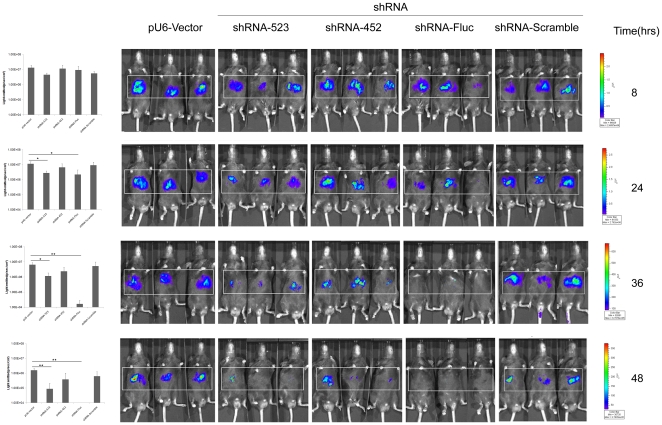
Efficient suppression of the HCV core protein expression by pU6- shRNA vector in the mouse liver. pU6-Vector, shRNA-Scramble vector, shRNA–Fluc vector, shRNA-452 vector or shRNA-523 vector (10 µg) were co-transfected with 10 µg pGL3-attB-CoreFluc vector into the mouse liver. Real-time *in vivo* imaging of FLuc activity in mice 8, 24, 36 and 48 hrs after hydrodynamic injection. The presence of statistical difference has been indicated as follows: *  =  *p*<0.05; **  = *p*<0.01. The area used for calculation of light-intensities has been boxed. All the experiments shown have been repeated at least thrice.

We proceeded to investigate whether two of these shRNAs employed in cell culture could similarly mediate a gene-silencing effect in adult mice by transient transfection, using real-time bioluminescence imaging. Four groups of mice were injected via the tail vein with 10 µg of pGL3-*attB*-CoreFluc and 10 µg of shRNA-Scramble, shRNA-452, shRNA-523 or shRNA-Fluc expression vectors respectively. Bioluminescence imaging was performed to examine luciferase expression in the liver at the indicated time after DNA injection. As illustrated in Figure 5, the effect of shRNA-Fluc and shRNA-523 was detectable as early as 24 h after transfection and became even more pronounced at later time points. By contrast, the effect of shRNA-452 and shRNA-Scramble was not detected until 48 h post-transduction.

### ΦC31 integrase mediates elevated transgene expression in the mouse liver

**Figure 6:**
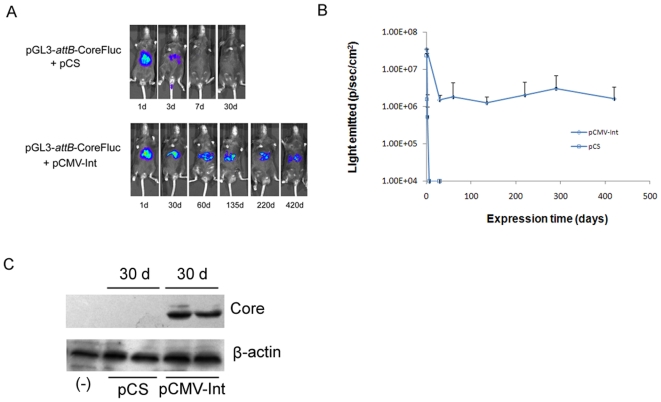
Integrase mediates increased and prolonged expression of HCV core protein and Fluc in the mouse liver. A.B. Bioluminescence imaging Fluc activity in mice. Two groups of mice received a large-volume tail vein injection of 10 µg of pGL3-*attB*-CoreFluc with 10 µg pCS(squares) or with 10 µg pCMV-Int(diamonds). FLuc activity was monitored by bioluminescence imaging in mice throughout the experiment. C. Western blot analysis of HCV core protein expression. Proteins were extracted from liver tissues and transferred to membranes, followed by incubation with monoclonal antibodies specific for the HCV core (top) and β-actin (bottom) that serves as a control for protein loading.

**Figure 7:**
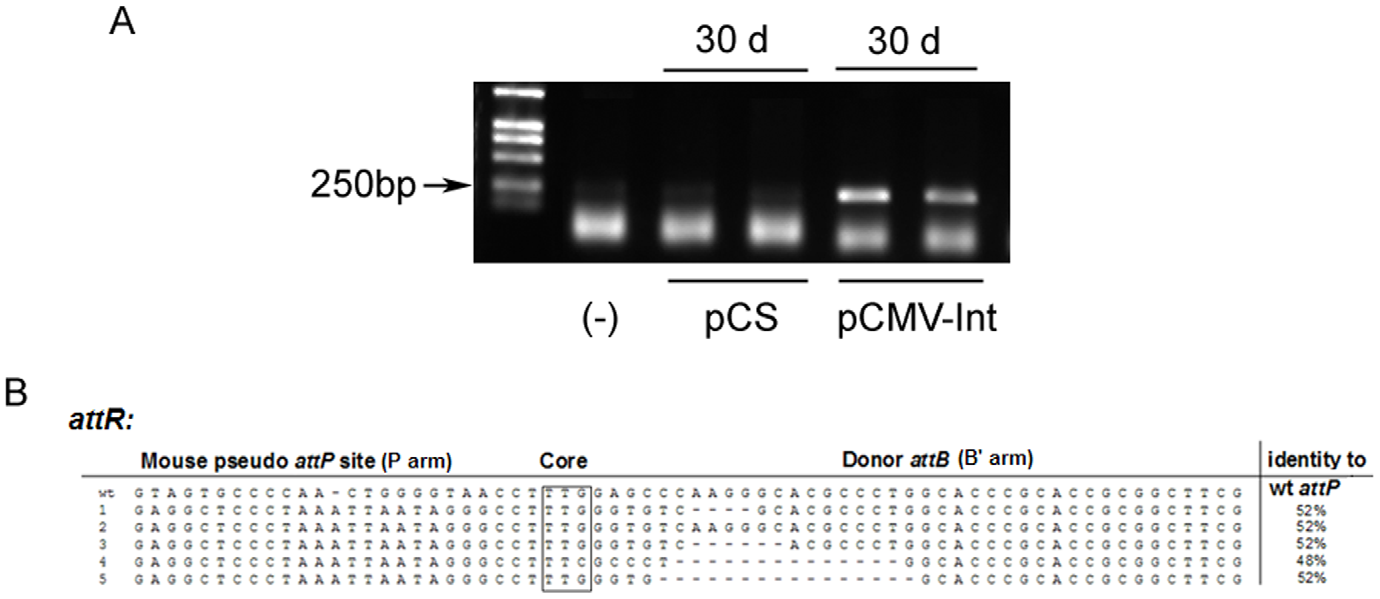
Detection of integrase-mediated site-specific recombination at the DNA level. A. Nested PCR was performed, using primers that detect the junction between the *attB* site of pGL3-*attB*-CoreFluc and mpsL1, a preferred pseudo *attP* site in the mouse genome. B. The sequences depicted the crossover region between the mouse pseudo *attP* site and the *attB* site. Shot lines represent bases missing in the novel joint. The 25 bp nearest the crossover of each genomic *P* arm was compared to wild-type *attP* [13].

Recent studies have demonstrated the successful use of ΦC31 integrase, which can catalyze the integration of plasmids into the mammalian genome at so-called “pseudo-*attP*” sites to achieve long-term gene expression [21]–[24]if those plasmids contain the attB recognition sequence. To determine the effect of ΦC31 integrase on the expression of the transgene, 10 µg of the pGL3-*attB*-CoreFluc was injected with either 10 µg of carrier plasmid pCS (control group) or the integrase expression vector pCMV-Int (test group) into the tail vein of mice. The luciferase activity was measured at different time points using the bioluminescence method. There was a high level of luciferase expression in the livers of all the mice 24 h after injection. When pCMV-Int was included, transgene expression decreased ∼30-fold within two weeks and lasted until day 420 (Fig. 6A and 6B), indicating that the integrase substantially increased and stabilized transgene expression. Mice from control group and test group were sacrificed 30 days post injection, and livers were removed from these mice. Total protein was isolated and western blot was done to analysis the HCV core protein expression (Fig. 6C). Genomic DNA was isolated, and genomic integration was confirmed by nested PCR (Fig. 7A). The resultant bands were sequenced and aligned with the genomic sites (Fig. 7B). The switch from *attB* to genomic sequence near the TTG core and the detectable sequence identity between the genomic sequence and *attP* confirmed ФC31-mediated integration at genomic pseudo-*attP* sites. These results further demonstrated that plasmid integration was associated with higher sustained levels of transgene expression.

### Core-shRNA-expression vectors inhibit HCV core gene and Fluc gene expression in the stable mouse model

As seen above, shRNA523 produced a higher inhibitory effect on the target gene, which was why shRNA523 was used for further studies in the stable mouse model.

**Figure 8:**
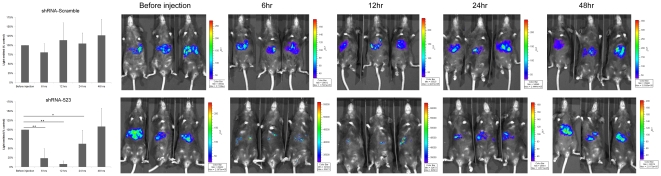
Efficient suppression of the HCV core protein and Fluc expression by pU6- shRNA expression vector in stable mouse model. shRNA-Scramble vector, or shRNA-523 vector (10 µg) were transfected into the stable mouse model. Real-time *in vivo* imaging of FLuc expression in mice 6, 12, 24 and 48 hrs after injection. The luciferase activity was plotted taking the control (before injection) as 100%.The presence of statistical difference has been indicated as follows: *  =  *p*<0.05; **  = *p*<0.01.

Three months after transfecion of pGL3-*attB*-CoreFluc and pCMV-Int, the mice were imaged with BLI system and divided into two groups. The mice were injected via the tail vein with either 10 µg/mouse of shRNA-Scramble expression vectors or shRNA523 expression vectors. We found that shRNA523 reduced luciferase levels by up to 76.4±26.0% (*P* = 0.00003) (n = 5, mean ±SD) and 91.8±8.0% (*P* = 0.00001) (n = 5, mean ±SD) compared to before injection, at 6 hr and 12 hr after injection respectively (Fig. 8). The inhibitory effect persisted for 1 day after a single injection while shRNA-Scramble did not seem to have any effect on the luciferase activity, suggesting the specificity of our method.

### Adverse effects of hydrodynamic injection shRNA-encoding plasmids on the mouse liver

**Figure 9:**
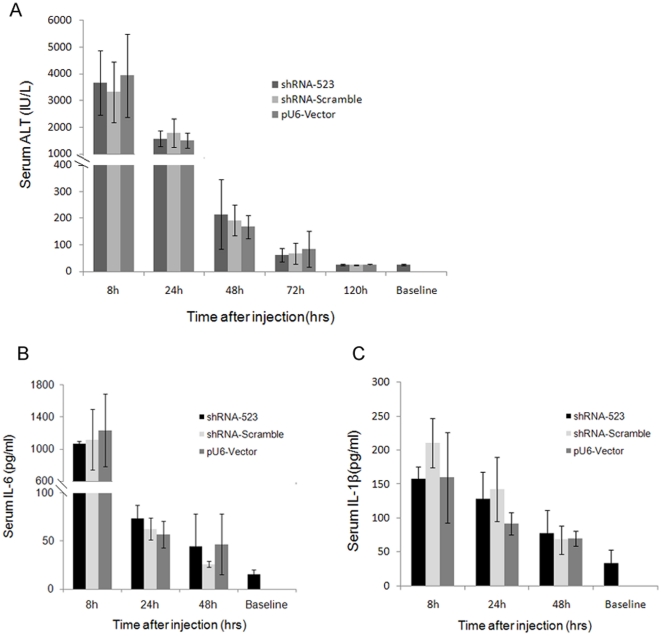
Hydrodynamic delivery shRNA effects on plasma ALT levels and cytokine IL-6/IL-1β levels in mice. C57BL/6 mice (n = 5 per group) were hydrodynamically transfected with 10 µg of shRNA-Scramble vector, or 10 µg shRNA–523 vector, or 10 µg empty control plasmid pU6-Vector. A. Plasma ALT and, B. plasma IL-6 and, C. plasma IL-1β levels (means ± s.d.) were determined at different time after transfection.

**Figure 10:**
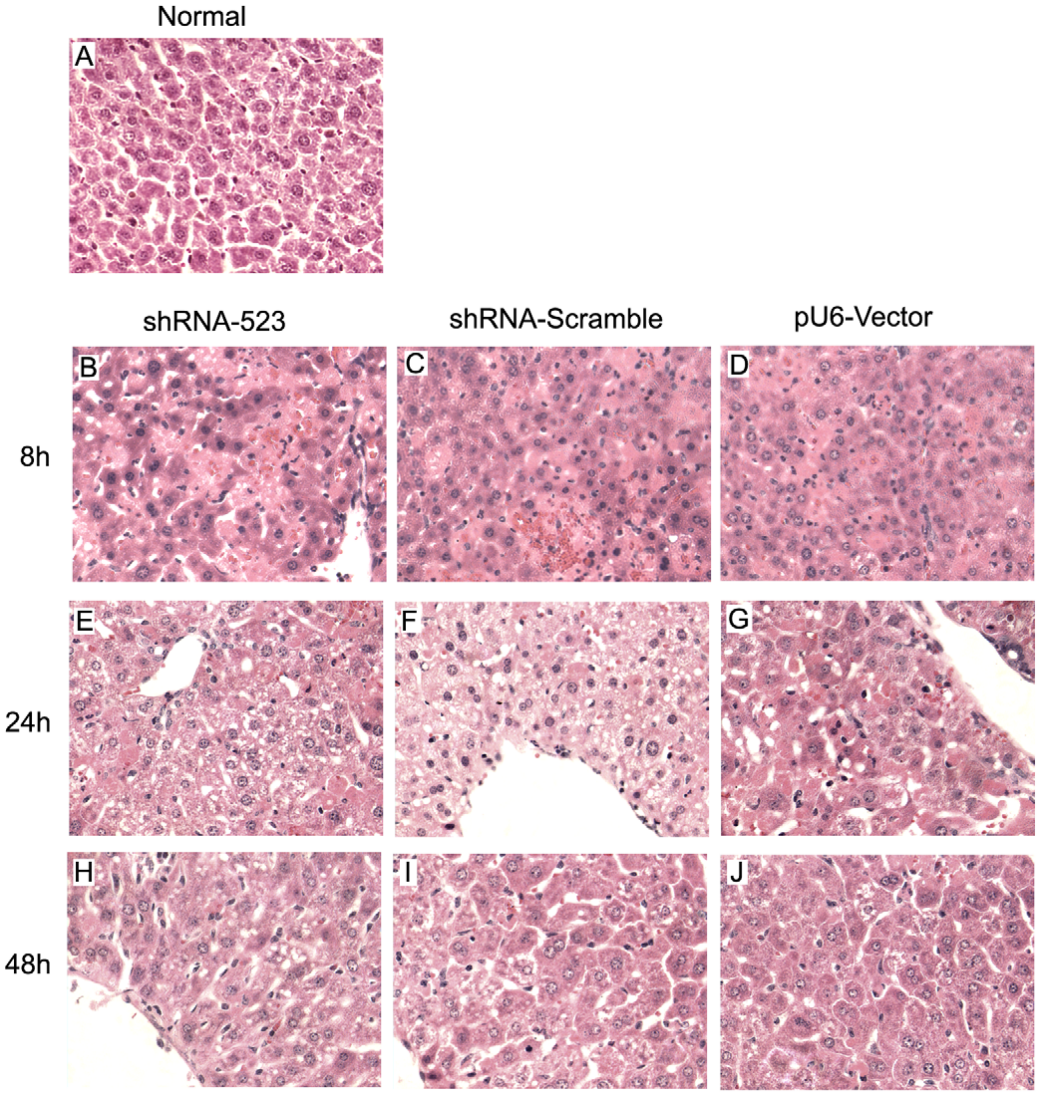
Effects of hydrodynamic injection shRNA on hepatic histology in mice. C57BL/6 mice were treated as described in the legend to Fig. 9. Liver sections were stained with hemotoxylin/eosin. Livers were examined histologically from normal, uninjected animals(A) or from animals sacrificed 8 h (B-D), 24 h (E–G), and 48 h (H–J) after hydrodynamic injection. Magnification, x 40.

To investigate the shRNA hepatotoxicity, the mice were injected with pSilencer-2.1-U6 plasmid, control non-targeting shRNA expression vectors (shRNA-Scramble), or shRNA523 expression vectors. Serum levels of alanine aminotransferase (ALT), a marker of liver function, were evaluated (Fig. 9A). ALT levels were significantly increased (3322–3932 IU/L) 8 h after injection, subsided to 167–214 IU/L by 48 h, then declined to the baseline by 120 h. There were no significant difference observed across all groups. In agreement with the ALT observations, cytokine IL-6 levels in serum, which is essential for an optimal acute-phase response after tissue damage, were very high (1065.67–1237.50 pg/ml) across each group 8 h post injection, subsiding to 26.00–46.87 pg/ml by 48 h, with no significant difference observed for shRNA-Scramble, shRNA523 vs. vehicle treatment(Fig. 9B). Another proinflammatory cytokine IL-1β levels exhibited a rise (157.25–211.00 pg/ml) 8 h after injection, followed by a return to the baseline levels during the next 48 hours (Fig. 9C). There was also no statistical significance between the groups. Examination of liver histology from both treated mice revealed significant hydrodynamic injection-related hepatic injury [25] (Fig. 10). At 8 h after injection liver morphology underwent remarkable changes. Many hepatocytes were swollen and their cytoplasm was vacuolized and stained less with eosin. Red blood cells (RBCs) appeared as clusters between and inside damaged hepatocytes. Cells developed signs of irreversible damage such as apoptosis or necrosis, accompanied by minimal neutrophil infiltration. Liver morphology 24 h after HTV injection was close to normal. Single cell necrosis, swollen cells and inflammatory infiltration were infrequent at 24 h, showing liver recovery at this time point. At 48 h the liver morphology became more normal. Taken together, these results indicated that liver damage observed in the mice was due to hydrodynamic injection, and all the mice could recover from hydrodynamic injection up to 2 days.

## Discussion

HCV genome is an ssRNA that functions as both an mRNA and a replication template so that destruction of HCV RNA could eliminate not only virally directed protein synthesis, but also viral replication. In cell culture, siRNA, as well as vector-encoded shRNA directed against the HCV viral genome, specifically targeting the HCV-5′-NTR, core, NS3, NS4b, NS5a and NS5b inhibits HCV gene expression [26]–[29]. RNAi represents a potentially promising approach to HCV infection, making it necessary to establish user-friendly and accurate non-primate systems for siRNA drug development.

We described here a novel method to screen anti-core protein siRNA in the mouse liver. By using the reporter gene, anti-core protein compounds can be screened by simply bioluminescence imaging the Fluc activity in whole animals under true physiological conditions.

In this study, three shRNAs targeting the highly conserved core region of the HCV genome and the plasmid pGL3-*attB*-CoreFluc, which encoded the fly luciferase (reporter gene) fusing to the downstream of HCV core protein as a silencing target, were co-transfected into Huh7 cells and the mouse liver. In cell culture, all the three shRNAs caused significant reduction in the level of HCV core protein while the sramble shRNA had no inhibitory effect on core protein expression. This observation had been previously reported by other groups. But Suzuki *et al* considered that shRNA452 construct mediated more effective inhibition of HCV replication than the other core-shRNAs (shRNA479 and shRNA523) [16], [17]. In our test, the inhibitory effects of these three shRNAs had no statistic difference. It was also found that the loss of Fluc activity coincided with the degradation of HCV core protein, which indicated that the Fluc activity could reflect the expression level of core protein successfully. In the transient mouse model, the inhibitory effect of shRNA452 and shRNA523 was examined by real-time bioluminescence imaging. The effect of shRNA-523 was detectable as early as 24 h after transfection and became even more pronounced at later time points. The effect of shRNA-452 was not detected until 48 h post-transduction.

There are some special requirements for assays used in drug discovery that are related to the nature of the information needed to understand drug action. Besides, advanced characterization of compounds typically requires answers to questions such as the relationship between duration of action and pharmacokinetics or the maintenance of efficacy after repeated dosing. So a stable mouse model can help to identify and evaluate specific compounds for their potential efficacy.

Phage ΦC31 integrase has emerged as a potent tool for achieving long-term gene expression in different tissues. Several studies document that phage ΦC31 integrase can site-specifically integrate plasmid DNA bearing an *attB* site into endogenous positions in the genome of mouse liver cells [21], [30], [31]. Using ΦC31 integrase, long-term expression of Core-Fluc was achieved. However, final expression values attained were substantially lower than the initial values at day 1 post-transfection. This is consistent with the findings of other groups and represents a transition from initial high levels of expression arising from unintegrated (episomal) pDNA to steady-state expression levels resulting from integrated pDNA[21], [24], [32]. In this stable mouse model, the inhibitory effect of shRNA523 was examined, and significant reduction in Fluc activity was observed. The inhibitory effect persisted for 1 day after a single injection.

Short hairpin RNAs (shRNAs) have emerged as a novel therapeutic modality, but there is increasing concern over nonspecific effects in vivo [33], [34]. Here, physiological effects of hydrodynamic injection of shRNA were detected in mice. Histological examination of livers at varying times post injection revealed initial hepatic injury at 8 h after injection that appeared to be fully resolved by 48 h.In agreement with the histology observations, serum ALT levels were significantly increased 8 h after injection, then declined rapidly within the next 48 hours, a finding that had been previously reported by other groups[19], [35]. Consistent with the ALT and histology observations, cytokine IL-6 and IL-1β levels, exhibited a dramatic rise 8 h after injection, followed by a return to the baseline levels during the next 48 hours. No significant difference was seen across mice transfected with shRNA523 expression vectors, non-targeting shRNA expression vectors or pSilencer-2.1-U6 plasmid. Altogether, our data suggest that liver damage observed in the mice is hydrodynamic injection method-related effects and transient shRNA synthesis has no detectable hepatoxicity. Given these findings, it may be important to consider background liver damage in the interpretation of gene knockdown via hydrodynamic injection shRNA. But proper experimental control can allow dissection of delivery-related side effects-mediated vs. gene knockdown- mediated changes.

In conclusion, a simple and quantitative method of real-time monitoring of HCV core protein inhibitors in mice has been successfully developed. Moreover, the method clearly demonstrates that shRNA targeting HCV core protein can effectively downregulate core gene and reporter gene expression in the liver of mice. This luminescence-based method allows continuous monitoring of the kinetics of HCV core protein inhibitors in live animals. This novel and simple method can be used for screening anti-HCV compounds.
